# Immersive virtual reality in orthopaedics—a narrative review

**DOI:** 10.1007/s00264-023-05911-w

**Published:** 2023-08-11

**Authors:** A. Combalia, M. V. Sanchez-Vives, T. Donegan

**Affiliations:** 1 Departament de Cirurgia i Especialitats Medicoquirúrgiques, Facultat de Medicina i Ciències de la Salut, Universitat de Barcelona (UB), c. Casanova, 143, 08036 Barcelona, Spain; 2 Servei de Cirurgia Ortopèdica i Traumatologia, Hospital Clínic de Barcelona, Universitat de Barcelona (UB), c. Villarroel, 170, 08036 Barcelona, Spain; 3 Facultat de Medicina i Ciències de la Salut, Universitat de Barcelona (UB), c. Casanova, 143, 08036 Barcelona, Spain; 4 Institut d’Investigacions Biomèdiques August Pi i Sunyer (IDIBAPS), c. Villarroel, 170, 08036 Barcelona, Spain; 5 Institución Catalana de Investigación y Estudios Avanzados (ICREA), Passeig de Lluís Companys, 23, 08010 Barcelona, Spain

**Keywords:** Virtual reality, embodiment, Pain, Orthopaedics, Post-surgical recovery, Rehabilitation

## Abstract

**Purpose:**

This narrative review explores the applications and benefits of immersive virtual reality (VR) in orthopaedics, with a focus on surgical training, patient functional recovery, and pain management.

**Methods:**

The review examines existing literature and research studies on immersive VR in orthopaedics, analyzing both experimental and clinical studies.

**Results:**

Immersive VR provides a realistic simulation environment for orthopaedic surgery training, enhancing surgical skills, reducing errors, and improving overall performance. In post-surgical recovery and rehabilitation, immersive VR environments can facilitate motor learning and functional recovery through virtual embodiment, motor imagery during action observation, and virtual training. Additionally VR-based functional recovery programs can improve patient adherence and outcomes. Moreover, VR has the potential to revolutionize pain management, offering a non-invasive, drug-free alternative. Virtual reality analgesia acts by a variety of means including engagement and diverting patients’ attention, anxiety reduction, and specific virtual-body transformations.

**Conclusion:**

Immersive virtual reality holds significant promise in orthopaedics, demonstrating potential for improved surgical training, patient functional recovery, and pain management but further research is needed to fully exploit the benefits of VR technology in these areas.

## Introduction

### Background and context

Orthopaedic interventions face many challenges including high costs, risks of complications, and long recovery times for patients. There is a need for innovative technologies that can improve the safety, effectiveness, and efficiency of orthopaedic procedures and outcomes. One such promising technology is immersive virtual reality (VR), which allows the creation and manipulation of realistic three-dimensional environments and virtual bodies. VR is already being used in a wide variety of fields including gaming, education, and healthcare but has only become more mainstream in the last few years due to huge advances in technology and dramatically reduced costs [1, 2]. The use of immersive VR and virtual embodiment in orthopaedics is still relatively new, but it is increasingly being used for training purposes, surgical planning, and improving the accuracy of surgery, as well as having significant potential for assisting with rehabilitation and recovery and helping to manage pain both during surgical procedures and post-surgically. According to a recent systematic review [3], VR applications in orthopaedic surgery have increased by 300% in the last decade and are expected to grow further in the near future.

**Fig. 1:**
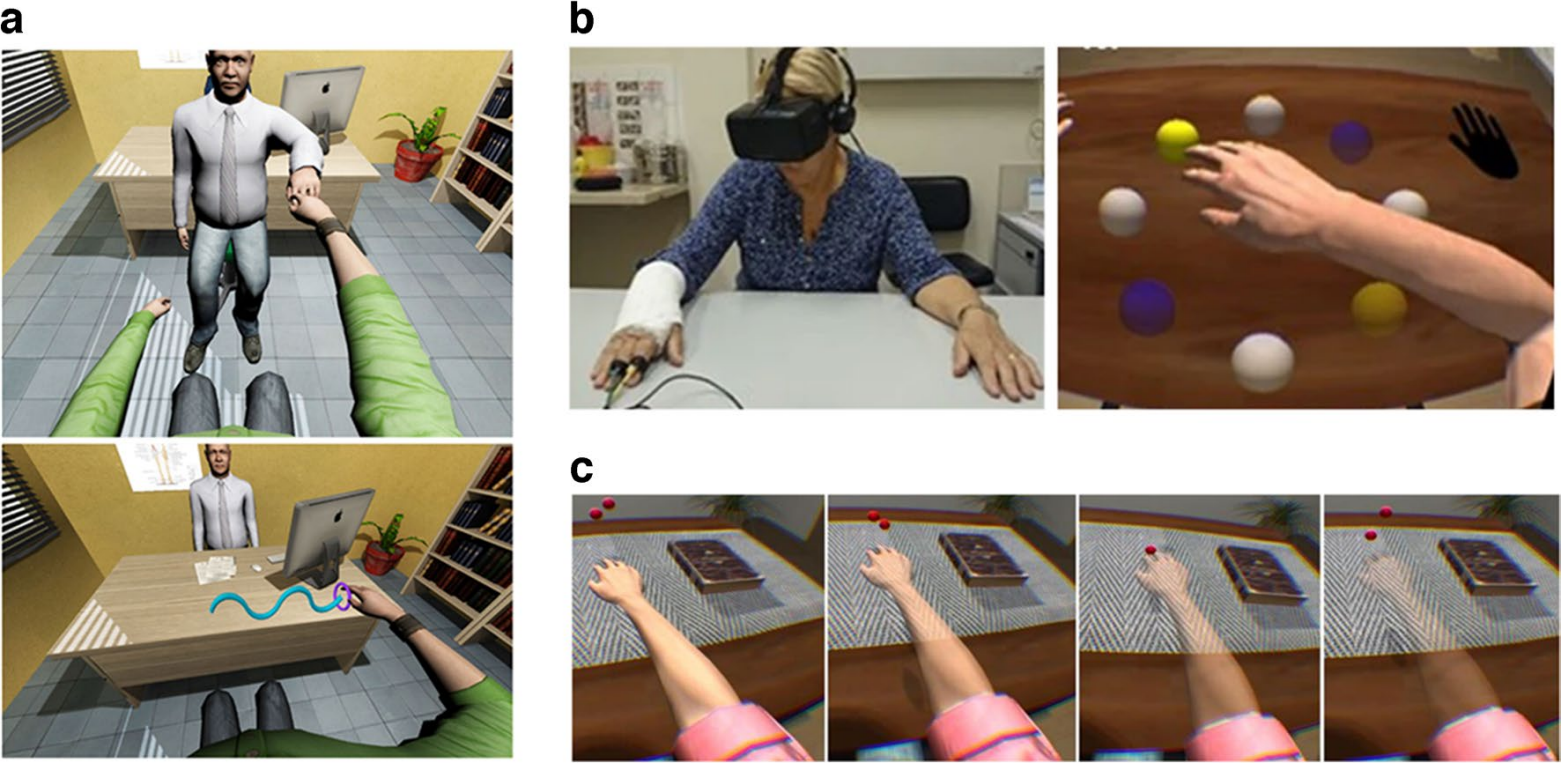
Virtual embodiment and first-person perspective for functional recovery and training. **a** Patient view from the head-mounted display. The body is seen from a first-person perspective. The panels in **a** represent a visit of the patient to a virtual hospital where he is examined and asked to perform a physical task. There is an interaction with a virtual clinician. This clinician could be entirely virtual or represent a real, remotely located person. Taken with permission from Perez-Marcos et al. (2012). *Frontiers in Neurology*, 3, 110. **b** Left panel, real-world view of the patient with the post-surgical, immobilized right arm. Right panel, first-person perspective of the arm performing a set of exercises following the process of embodiment. Taken with permission from Matamala-Gomez et al. (2022). Impact of virtual embodiment and exercises on functional ability and range of motion in orthopedic rehabilitation. *Scientific Reports*, 12(1), 5046. **c** Approaches to the reduction of chronic pain: varying transparency of the embodied arm. The red ball is used for visuotactile multisensory stimulation in order to induce ownership over the virtual arm. Taken with permission from Matamala-Gomez et al. *The Journal of Pain* 20.6 (2019): 685-697

VR refers to a computer-generated simulation or environment that immerses users in a three-dimensional, interactive experience, typically involving sight, sound, and sometimes touch, creating the illusion of being physically present in a virtual world. VR users typically wear a head-mounted display (HMD) that covers their eyes and ears and use handheld controllers or gloves to interact with the virtual environment. VR can create a sense of presence, which is the feeling of being physically located in the virtual environment, and immersion, which is the degree to which the virtual environment blocks out or replaces sensory information from the real world [4, 5]. It is also possible to give the user a virtual body with which to explore the virtual environment, over which the user can have a sense of agency and control [6]. Using motion sensors and cameras, movement of the user’s real body can produce simultaneous corresponding movement of the virtual body (visuomotor congruence [7]), and virtual objects seen to touch the virtual body can be felt simultaneously at the corresponding point on the real body (visuotactile congruence [8]) using haptic feedback. This congruent multisensory stimulation can produce a feeling of ownership and agency over the virtual body—a phenomenon known as virtual embodiment. Once the virtual body is perceived as one’s own, changes or experiences affecting the virtual body have been shown to impact the real body across a variety of physical and psychological measures, including pain perception; for example, alterations in color, transparency levels, or size of a virtual arm have all demonstrated effects on pain thresholds in both healthy individuals and chronic pain patients [9, 10] (Fig. 1c).

Fully immersive VR is distinct from, but often confused with, other related technologies such as augmented reality (AR) and mixed reality (MR) and non-immersive VR. AR is a technology that overlays digital information or images onto the real world, creating a mixed reality that enhances or augments the user’s perception of their surroundings. AR users typically wear transparent glasses or use smartphone cameras to see the digital content superimposed on their view of the real world. AR can be used to provide additional information or guidance to users without isolating them from their physical environment [11]. MR, on the other hand, goes beyond AR by integrating virtual objects into the real-world environment and allowing users to interact with both the physical and digital elements in a more immersive and interactive way. MR enables virtual objects to interact with the real world and vice versa. Immersive VR, AR, and MR can be also referred to under to broader term of XR (extended reality). Non-immersive VR refers to technologies that use two-dimensional screens or monitors to display virtual environments or simulations, without blocking out the real world or creating a sense of presence or immersion, and in which first-person perspective or full embodiment cannot be realized. AR, MR, robotic applications, and non-immersive VR are beyond the scope of this review, and we will focus more directly on fully immersive VR and virtual embodiment.

This narrative review aims to shed light on recent advancements and future directions of immersive VR and virtual embodiment in orthopaedic pre- and peri-surgical settings, and in post-surgical recovery. We will also consider how the recent extraordinary advances in AI can be used in conjunction with VR to enhance the personalization and adaptation of VR interventions. By examining the potential of VR and embodied virtual bodies, we aim to uncover promising avenues for improving the safety and effectiveness of orthopaedic treatments as well as helping to optimize patient recovery post-surgery. The review is organized as follows: first, we will discuss the advantages of VR to surgeons when used pre- and peri-operatively, and to patients when used before or during surgery for pain relief and reduction of anxiety. Second, we will discuss the use of virtual embodiment in post-surgical recovery, in particular recent studies that have investigated the effects of VR and virtual embodiment on pain, anxiety, mobility, and function in post-orthopaedic surgery patients. Third, we will discuss some of the challenges and limitations of VR and virtual embodiment in orthopaedics, as well as some possible solutions and recommendations. Finally, we will conclude with some implications and directions for future research and practice.

## Pre- and peri-surgical uses of immersive VR

### For surgeons

In recent years, immersive VR has been increasingly utilized in surgical education, with applications in anatomy instruction, surgical skills training, intraoperative communication (for example with remote surgical supervision), and preoperative planning. Immersive VR can be used to teach anatomy to surgeons by providing a realistic and interactive 3D environment that allows them to practice surgical procedures and explore anatomy in detail [12, 13]. The ability to manipulate, add, and remove anatomical layers and individual structures in three dimensions confers a significant advantage over learning from 2D images. Many commercially available VR applications allow the user to visualize, interact with, and edit 3D human anatomy models, as well as medical imaging and human dissections [14]. Moro et al. [15] compared VR, AR, and flatscreen apps for learning anatomy in a randomized study and found that members of the AR and VR groups reported increased learner immersion and engagement, but without a significant difference in the assessment score between the groups.

Immersive VR can be used for surgical supervision by allowing remote experts to guide and monitor trainees or junior surgeons in real time. This can enhance the quality and safety of surgical care, especially during emergencies or in situations where resources are limited [16]. However, immersive VR simulation training may also induce a higher cognitive load and result in poorer performance than conventional VR simulation training in laparoscopy [17].

Surgical training platforms that leverage VR can provide immersive education for students and surgeons to evolve the skill sets required for advanced medical procedures, and immersive VR is being used to train surgeons by providing them with a realistic environment in which to practice procedures. The combination of increasingly life-like visuals and tactile feedback help make experiences comparable to real surgery, and the visuospatial skills acquired through immersive VR training have significant potential to translate to the operating room. The incorporation of immersive VR into surgical training programs is supported by high-quality, albeit heterogeneous, studies demonstrating improved procedural times, task completion, and accuracy, positive user ratings, and cost-effectiveness [18].

Using pre-surgical imaging, immersive VR can provide a realistic and immersive representation of the individual patient’s anatomy and pathology by converting 2D images from CT or MRI into interactive 3D models. This can help a surgeon’s understanding of anatomy and the anatomical variation between patients (the vascular system in particular) and allow them to plan and perform procedures more accurately and efficiently. In addition, a more precise alignment and positioning of implants and instruments can be facilitated, which may help to reduce errors, revisions, and infections, as well as improve functional outcomes and patient satisfaction [19]. Indeed, a recent scoping review found that the pre-operative use of immersive VR systems resulted in reduced operation times, reduced inadvertent damage to neighbouring tissue, reduced blood loss, and shorter hospital stays [20].

Clearly, because the user’s worldview is completely restricted when using immersive VR, it generally serves no useful purpose for the surgeon during the surgery itself. AR and MR, on the other hand, allow the highly useful direct visualization of surgical data such as imaging and anatomical landmarks into a single view during surgical procedures. The use of these technologies is beyond the scope of this review but is reviewed in detail elsewhere [21].

### For patients

Orthopaedic surgeries and procedures are often associated with pain, anxiety, and discomfort. Traditional methods of pain management, such as opioids and other medications, have their limitations and come with significant risk and side-effect profiles, while anaesthetizing patients can occasionally result in complications such as oversedation, post-surgical dementia, and death. The prescription opioid epidemic [22] has contributed to the interest in other approaches, such as the so-called Digiceuticals, which include the use of VR. This is one of the reasons why there has been a growing interest in the use of immersive VR therapy as a non-pharmacological approach to help manage pain and anxiety in orthopaedic patients. Patients can be provided with positive or relaxing stimuli, such as music, games, or nature scenes, for example. VR may work by distracting the patient’s attention from the painful or stressful stimuli—it is thought that the sense of being present in the virtual world occupies significant sensory “bandwidth”, reducing the availability for nociceptive processing [23].

The concept of peri-procedural VR as a distraction-based analgesic method has been in existence for over 20 years, since Hunter Hoffman first implemented using VR with adolescent patients with severe burns, who experienced a virtual ice and snow-filled landscape while having their dressings changed, a notoriously painful procedure [23]. The potent analgesic effect of this type of intervention for burn patients, as well as its positive effects on perioperative anxiety and stress, has repeatedly been demonstrated in high-quality trials (see [24]) and is largely based on distraction by full immersion in engaging and interactive environments.

For orthopaedic surgeries and procedures, there is more limited evidence for positive effects concerning peri-procedural analgesia, with fewer controlled trials and heterogeneous procedures and outcome measures.

In a randomized controlled trial [25], 98 patients requiring hand dressing changes were randomized to a VR or a non-VR control group before the procedure. Those in the VR group reported a highly significant improvement in pain post-procedure compared to controls (mean improvement of 3.89 on an 11-point visual analog scale, versus a worsening of 1.15). Not all studies have shown positive results. In another randomized study by Walker et al. [26], the authors evaluated the effect of a VR game on pain and anxiety during flexible cystoscopy in adult men. Subjects in the VR group had to shoot snowballs at various targets while immersed in a snow-filled landscape, while those in the control group observed the operation on a monitor. They found no significant difference between the control group and the VR group in terms of pain, anxiety, vital signs, and galvanic skin response. They concluded that VR distraction did not provide any benefit for alleviating pain and anxiety during cystoscopy in this population. These results may be partly explained by the relatively lower levels of pain and anxiety in the population compared with previous studies.

VR may also be of therapeutic use in reducing pre-surgery anxiety and post-surgical anxiety and pain. A recent systematic review concluded that VR can reduce preoperative anxiety scores and improve haemodynamic parameters in patients undergoing surgery, especially in paediatric patients [27]. Post-operatively, in a pre-post experimental case series of patients undergoing cardiac surgery [28], researchers explored the effect of a 30-min VR intervention designed to reduce postoperative pain and anxiety (contemplation in a variety of relaxing landscapes) given within 24 h of cardiac surgery. They found that 88% of patients reported a decreased level of pain post-VR; 37% had a lower heart rate; 52% had reduced mean arterial pressure. In contrast, a recent RCT examined the use of VR and hypnosis (individually and combined) in patients recovering from cardiac surgery and found no difference between any of the groups [29].

## Post-surgical uses of immersive VR

### Why VR?

In many areas of rehabilitation, the integration of exercises performed by virtual bodies has been extensively employed, particularly in stroke rehabilitation [30] (Fig. 1a) and treatment for phantom limb pain (see [31] for a review). The application of virtual bodies in orthopaedic post-surgical recovery, however, remains relatively unexplored, despite many potential advantages. Currently, VR in rehabilitation is most commonly used as an adjunct to conventional therapy, and more rigorous research and larger samples are required for a consistent demonstration that physical functional recovery in virtual settings is superior to conventional therapy on its own. However, a recent systematic review found that VR exercise has the potential to exert a positive impact on individual physiological, psychological, and rehabilitative outcomes compared with traditional exercise [32], although the quality, quantity, and sample size of existing studies meant that drawing firm conclusions was difficult. VR for physical rehabilitation is considered low-risk and beneficial in most studies, with no adverse events after VR rehabilitation being reported [33, 34].

Physical recovery mediates its effects on tissue through mechanical loading but also makes use of functional plasticity in the cerebral cortex, with sensorimotor training influencing cortical remodeling for example. The importance of targeting neuroplasticity as part of rehabilitation is becoming increasingly recognized (e.g., [35]) and will likely form a core component of future functional recovery approaches for many injuries. But where does VR fit in? What can a digital environment provide that rehabilitation in the real world cannot? An important component of movement consists of movement or repetition of tasks, but what difference does it make when repeating movements in the real or digital world? Some reasons that make virtual environments useful in rehabilitation include:

1. The nature and pattern of feedback provided to the patient can be changed in real time. This feedback can be altered or enhanced across different sensory modalities to better recruit sensorimotor circuits and increase the effectiveness of rehabilitation. This may be particularly useful in highly fearful patients (or patients with extreme pain with movement) who can initially experience their virtual selves moving and exercising, but without moving in reality [36, 37]. Fear avoidance is a significant barrier to adherence, and VR can effectively address this problem through treatment techniques such as graded exposure (see e.g., [38])
2. Recovery from orthopaedic interventions is often long and arduous, and adherence to rehabilitation interventions is required to optimize recovery time. Poor adherence to exercise programs is a significant barrier to recovery and is thought to generally vary between 40% and 50% [39]. Poor adherence may be related to situations where the program interferes with everyday life [40], cognitive factors such as kinesiophobia [41], and the exercise not being tailored well enough to the patient, particularly in group settings [42], or simply a lack of patient motivation. Repetitions of movements are often tedious, and a patient’s motivation can deteriorate rapidly, especially since the positive effects of rehabilitation, which act as rewards, are rarely immediate. Virtual environments can provide more engaging and motivating contexts for rehabilitation, including gaming approaches. They can also provide ecologically valid, accurately measurable, and salient tasks that can be more motivating than mindlessly repetitive movements. This can help dramatically improve adherence and such approaches have been shown to significantly improve patient motivation and enjoyment in virtual rehabilitation [43]. Gamification of functional recovery tasks also helps to improve patient motivation and results in improved outcomes across a wide variety of musculoskeletal conditions [44].
3. The patient’s performance during rehabilitation can be tracked in real time, with the exercise being modified according to performance, and the information can be recorded and provided to the healthcare professional who can be located locally or remotely [30]. This allows the patient to perform their rehabilitation at a time and place of their choosing and removes a significant barrier to exercise adherence. Remote movement tracking in VR is in the very early stages of development but has been preliminarily explored in other patient groups (e.g., [45]).
4. Interaction with the virtual environment via the virtual body can be mediated not only through motor activity but also through other means such as brain-computer interfaces [46, 47]. This has potential implications for therapeutic interventions aimed at patients with an inability to move, for example through immobilization or paralysis.

### Immobilization

Immobilization can be considered a necessary evil in recovery from injury or surgery. While necessary for some tissues to heal, it simultaneously reduces muscle strength, power, and endurance, induces atrophy, increases joint stiffness, and impairs proprioception [48]. There is significant evidence that many of these impairments are largely neurally mediated (e.g., through corticospinal inhibition), since motor imagery practice (strongly visualizing a movement without performing it) during a period of immobilization significantly ameliorates these effects [49]. This opens a significant therapeutic avenue for immersive VR and specifically virtual embodiment. For patients, being embodied in a virtual body means that they can plan, visualize, and then experience their virtual body moving and exercising without actually moving themselves—a combination of both motor imagery and action observation, which have been shown to have their effects through separate neural pathways [50]. If patients experience a strong illusion of their limb moving, could this reduce some of the negative effects of immobilization? To try to answer this question, Matamala-Gomez et al. [37] recently investigated how virtual embodiment would improve motor functional ability and accelerate the functional recovery process in patients with a distal radius fracture of the arm who were immobilized for several weeks (Fig. 1b). They conducted a randomized controlled trial with 54 patients who were assigned to either an immersive VR group, a non-immersive VR group (who observed the exercises on a flat screen), or a conventional digit mobilization group. They found that the immersive VR group showed greater improvement in functional ability and range of motion than the other two groups, and that these outcomes were correlated with the degree of ownership and agency over the virtual arm. They also found that the immersive VR group reported lower pain and disability scores than the other two groups. The authors suggest that via repeated activation of the neural networks involved in action planning, motor imagery, and action observation, detrimental neuroplasticity changes occurring in the cortex are minimized. The authors also speculate there may additionally be a descending impact on the autonomic system and muscles, enhancing heart rate, respiratory rate, and skin and muscle blood flow through cholinergic vasodilation, which helps reduce the effect of immobilization. The results support the idea that immersive VR based on embodiment and exercises can enhance the functional recovery process of immobilized patients by providing a more engaging and motivating experience, simulating real-life situations, reducing pain and stress, and improving motor function.

### Movement-induced pain

Movement-induced pain is another potential barrier to recovery and is associated with kinesiophobia in many orthopaedic conditions [51]. Here as well, illusory movement in VR may be of benefit, especially when used as part of a graded exposure approach. In a pre-post experimental study [52], 24 patients with movement-related shoulder pain were exposed to a single session of VR therapy in which they were embodied in a virtual body and observed their virtual arms performing movements and exercises that were difficult and painful to do in real life. The researchers measured patients’ pain-free active range of movement before and immediately after the 15-min intervention and found clinically and statistically meaningful improvements in pain-free abduction and hand-behind-back ranges of movement. The results are consistent with the idea that violating expectations (in this case, not experiencing pain when a limb is seen to be moving into a previously painful range) is sufficient to modulate pain in real-life movement. This is consistent with another VR study showing that a pain-free range of motion in the cervical spine can be modulated by visual feedback [53], although the findings are not always consistent (cf. [54]). Further trials with repeated and progressed treatment are required to determine longer-term clinical efficacy.

### Reducing reinjury

The very high reinjury rates associated with some injuries and surgeries such as ACL reconstruction are another major problem in orthopaedics. Post-surgical functional recovery strategies that focus largely on recovery of traditional outcomes such as range of motion, strength, and balance may be insufficient, since risk factors for reinjury may involve the neurological response to the injury and/or surgery and not just the physiological and biomechanical changes of the knee [35]. A combination of disrupted afferent and reduced efferent neural signaling is thought to lead to arthrogenic muscle inhibition and motor control changes, with resulting neuromuscular compensations that may place the patient at a higher injury risk [35]. To address this altered neurological response, there have been recent calls to integrate a far wider variety of motor relearning strategies, with a significant variation in attentional and environmental factors that more closely replicate real-world scenarios [55]. Immersive VR can have a significant role to play here, particularly in the field of sports. Game-like situations with environmental aspects such as opposing players, crowd noise, and weather can easily be reproduced in VR and provide safe yet challenging environments for rehabilitation. Such experiences can also be highly motivating for the patient, which is particularly useful for longer-term rehabilitation. While there are few high-quality studies to date exploring virtual lower limb functional recovery (just two in a recent systematic review; [56]), there is preliminary evidence supporting the superiority of using VR for balance outcomes [57, 58], and improving perceptual-cognitive skills such as visual search and decision-making, enhancing psychological resilience, and improving mental performance under pressure in healthy football players [59, 60]. Whether this translates ultimately to reduced injury or reinjury rates remains to be elucidated.

## Challenges and limitations of VR in orthopedics

### Equipment and costs

Historically, VR required sophisticated hardware that was expensive, complex, cumbersome, and prone to malfunction or failure. Recent advances in both hardware and software have alleviated many of these problems. Self-contained wireless VR setups from different makers are available for a few hundred euros, down significantly from the thousands of euros only a few years ago. Software development costs, until very recently, have also been high, since customization of virtual functional recovery programs is highly labour-intensive. Indeed, many VR clinicians and researchers have simply used commercially available applications and games for this reason. However, powerful yet user-friendly development software such as “Unity®” has made programming applications significantly more accessible than previously, and this will only improve with the recent advances in AI (discussed below).

### Cybersickness

Cybersickness can be an occasional problem in VR, in particular in patients with a lower threshold for dizziness or nausea [1] and in environments with events at high speeds (e.g., rollercoasters), but recent technological advances have ameliorated many of these problems. Similar to motion sickness, cybersickness is triggered by a disconnect between the visual and vestibular system—when the brain perceives movement in the virtual environment, but the body remains stationary, and can lead to symptoms such as nausea, dizziness, headaches, and general discomfort. With continuing improvements in display resolution, frame rates, and latency, cybersickness is less likely to be problematic. In addition, the use of airflow [61], narrowing the field of view [62], and altering the interpupillary distance (IPD) [63] may also help. For users, graded exposure with frequent breaks will be of benefit, as will focusing on a stable object away from the screen or the horizon.

### Ethical and legal implications of VR in orthopaedics

VR users may generate personal data, such as biometric, health, and location data, which have the potential to be accessed or misused by third parties without their knowledge or consent. As with other personal data, VR-acquired data should follow existing regulations and ethical considerations [18, 58]. Clear policies and standards for data collection, processing, and sharing in VR application, as well as educating and empowering users to make informed choices and exercise control over their personal data in VR environments, are required, as discussed in detail by Madary and Metzinger [64].

### VR clinical trials standardization

While VR still lacks standardized guidelines, protocols, and criteria for evaluation and implementation in clinical practice, from a research perspective, there have been recent attempts to develop a research framework for studying the therapeutic effects of VR. A working group of more than 20 international VR experts developed a methodological best-practice framework [65]. Three phases of VR clinical study designs are recommended—the first phase focuses on content development by working with end users using the principles of patient-centered design. The second phase involves beta testing with a focus on feasibility, acceptability, tolerability, and initial clinical efficacy with testing taking place in the intended clinical environment. Finally, randomized, controlled studies that evaluate efficacy against a control condition should be carried out. The framework emphasizes the critical importance of end-user feedback in the development of VR interventions before expensive large-scale trials are carried out (see [66])

## AI and integration with VR

VR application development is likely to become significantly easier with the recent dramatic advances in AI. The creation of fully navigable and customizable 3D virtual environments and avatars will soon be considerably faster and require less programming knowledge than it used to be. This is likely to open a new realm of possibilities for VR applications in many domains including health care. AI can also enhance the interactivity and realism of VR experiences by enabling natural language processing, computer vision, and machine learning capabilities. These can allow VR systems to understand and respond to user inputs, gestures, emotions, and preferences in a more human-like and adaptive way. For example, machine learning techniques can be used to personalize the exercise program and make it more appropriate for the individual patient. Using features such as eye tracking [67], facial expression recognition [68], or more simply pain report or self-perceived effort, algorithms can adjust the dosage and type of exercise in real time. This automated personalization may help to remove a significant barrier to adherence often seen in group settings where a one-size-fits-all approach is very common. Machine learning can also be used to drive intelligent guidance, coaching, and motivation to the patient, using natural language processing, speech synthesis, and conversational agents, such as virtual clinicians, physiotherapists, or other patients.

### Conclusion

Immersive VR is a promising technology that has the potential to transform orthopaedic practice and improve patient outcomes. While further research is needed to fully understand the benefits and limitations of VR therapy, the growing body of evidence suggests that it is a promising approach for enhancing pre-surgical and peri-surgical education and training for patients and surgeons, facilitating post-surgical functional recovery, alleviating immobilization-related stress and boredom, and reducing movement-induced pain and injury without the use of medication. Additionally, VR provides immersive and engaging environments for exercise and therapy. However, VR also faces some challenges and limitations, such as the availability and affordability of equipment and software, the risk of cybersickness, the ethical and legal implications of VR use and data collection, the lack of standardized methods and protocols for VR clinical applications, and the need for more evidence-based studies to evaluate the efficacy and safety of VR interventions. Furthermore, VR can benefit from the integration of AI techniques, such as machine learning, computer vision, natural language processing, and affective computing, to create more personalized, adaptive, and interactive VR experiences that can respond to the user’s needs, preferences, emotions, and feedback. Future research should explore the synergies between VR and AI, as well as address the gaps and challenges in the current state of VR in orthopaedics.
